# Enhancing Fire Resistance of Geopolymers Modified with Thermal Insulation Additives

**DOI:** 10.3390/ma17194854

**Published:** 2024-10-02

**Authors:** Maja Kępniak, Jakub Zabawski, Piotr Prochoń

**Affiliations:** Department of Building Materials Engineering, Faculty of Civil Engineering, Warsaw University of Technology, Armii Ludowej 16, 00-637 Warsaw, Poland; jt.zabawski@gmail.com (J.Z.); piotr.prochon@pw.edu.pl (P.P.)

**Keywords:** geopolymer, fire resistance, thermal resistance, fly ash, perlite sand

## Abstract

This study aims to improve the fire resistance of geopolymers by adding thermal insulation materials. These additives help the material perform better at high temperatures. Previous research focused on using fly ash, metakaolin, and zeolite in geopolymer composites. This study looks at how porous additives affect compressive strength and whether non-destructive testing can measure damage after heat exposure. Four temperature tests were set: 400 °C for 60 min, 400 °C for 120 min, 800 °C for 60 min, and a maximum of 658 °C for 120 min. The results showed that the compressive strength and ultrasonic pulse velocity (UPV) dropped as the temperature increased, with a sharp decrease at 800 °C. Unmodified samples broke apart at high temperatures, while modified samples lost 40% to 70% of their strength. The study confirmed that a dense, amorphous matrix improves heat resistance, even with porous additives like fly ash. A link between UPV and compressive strength was found, suggesting non-destructive testing could be useful for checking structural integrity after a fire.

## 1. Introduction

In recent years, the construction materials industry has increasingly prioritized sustainable production methods, focusing on the development of composites with a reduced carbon footprint [1,2]. Among these, alkali-activated materials, such as geopolymers derived from fly ash, have garnered significant attention [3,4,5]. Geopolymers offer numerous advantages, including high strength [6], durability, low permeability, and resistance to chemical attack [7,8,9]. Unlike conventional cement-based binders, they also have a considerably lower carbon footprint, as their production requires less energy and emits significantly less carbon dioxide [10,11].

In the context of striving for sustainable development, there is increasing interest in low-carbon construction materials and those incorporating waste products. However, the durability of structures and user safety remain crucial factors. Some studies on eco-concretes, such as those with recycled aggregate [12] or calcium sulphoaluminate cement [13,14], indicate a decline in high-temperature resistance, especially in the 400–600 °C range. On the other hand, certain ecological modifications, such as the addition of fly ash [15,16] or ground granulated blast-furnace slag [17], have been shown to improve fire resistance, particularly by reducing spalling. Replacing 40% of the cement mass with these materials helps maintain strength up to 400 °C. Against this background, concretes based on alkali-activated binder systems, which are more stable at high temperatures and less prone to spalling, present a promising option for use in evacuation routes and other critical infrastructure. Another aspect related to sustainability is the long-term durability of materials, especially under repeated fire or thermal cycling. In this context, the use of geopolymers is highly promising. Research shows that fly ash-based geopolymeric mortars remain stable after thermal treatment, retaining acceptable compressive strength even at elevated temperatures [18,19]. Moreover, their thermal stability after repeated cycles suggests that these materials are suitable for applications like thermal energy-storage concretes [20].

One of the distinguishing features of geopolymers, particularly in comparison to traditional construction composites, is their remarkable resistance to elevated temperatures. Conventional concrete, when subjected to high temperatures, such as those encountered during a fire, often undergoes degradation. The calcium hydroxide formed during the hydration process can decompose, leading to a breakdown of the microstructure and a loss of mechanical properties [21,22]. In contrast, geopolymers exhibit excellent high-temperature resistance, largely due to their unique polycondensation reaction mechanism, which differs fundamentally from the hydration process in Portland cement [23,24]. The precursor in geopolymer concrete plays a crucial role due to its high chemical reactivity, forming robust and resistant materials when combined with alumina silicate-rich substances like GGBFS [25], fly ash [26], rice husk [27], or metakaolin [28]. Activator solutions composed of sodium or potassium silicate and hydroxide in varying ratios and molarities effectively disturb and activate the glass phase, leading to a hydration process that produces calcium aluminosilicate hydrate (CASAH) gel [29]. This process results in a high aluminum content, with CASAH’s amorphous microstructure contributing significantly to strong bond formation and overall material density [30]. Therefore geopolymers have enhanced fire resistance and reduced reductions in compressive strength, cracking, and spalling [23,31].

The unique fire resistance of geopolymers enables them to maintain stability at temperatures exceeding 1000 °C, making them suitable for applications requiring high thermal resistance [32]. Their low thermal conductivity, minimal shrinkage and cracking under heat, and retention of mechanical properties even after prolonged exposure to high temperatures further enhance their appeal as a material for fire-resistant construction [33]. Additionally, their fire resistance—characterized by an ability to withstand high temperatures without igniting or releasing toxic fumes—makes them a valuable material in the context of construction, where fire safety is a paramount concern [34]. However, the performance of geopolymers at elevated temperatures is not solely dependent on their inherent properties; it is also influenced by factors such as composition, curing conditions, and the specific environment in which they are applied [34].

Geopolymer concrete has demonstrated remarkable thermal resilience, particularly at the microscale, showing reduced cracking, delamination, and spalling when exposed to high temperatures. Research on fly ash-based geopolymer concrete highlights its superior performance compared to ordinary Portland cement (OPC) concrete, particularly in maintaining strength and stability at temperatures up to 1000 °C. The enhanced fire resistance of geopolymer concrete is attributed to the development of strong alumina silicate networks and a refined pore structure, which both improve its thermal and mechanical properties. The addition of certain fillers, such as basalt and ceramic materials, further enhances these properties. Additionally, color changes in geopolymer concrete at high temperatures can aid in postfire assessments. The material’s potential applications include fireproof panels, coatings, and other fire-resistant construction elements, making it a promising alternative for structures exposed to high-temperature environments.

Moreover, despite their advantages, geopolymers present certain challenges, including complex production processes and shorter workability times compared to cement composites [35,36]. Furthermore, while the precursors used in geopolymer production are often waste materials or industrial by-products, the alkaline activators involved can have a significant environmental impact due to their high carbon footprint and potential health risks.

Given these characteristics, the goal of this study is to enhance the thermal insulation properties of geopolymers while maintaining their high-temperature resistance. Previous research has explored the design of geopolymer composites using a combination of fly ash, metakaolin, and zeolite precursors, modified with various porous additives intended to improve thermal insulation [37,38]. The current study builds on this work by evaluating the effects of these modifications on compressive strength and exploring the feasibility of using non-destructive methods to assess the strength degradation of geopolymer composites after exposure to elevated temperatures.

## 2. Materials and Methods

**Figure 1:**
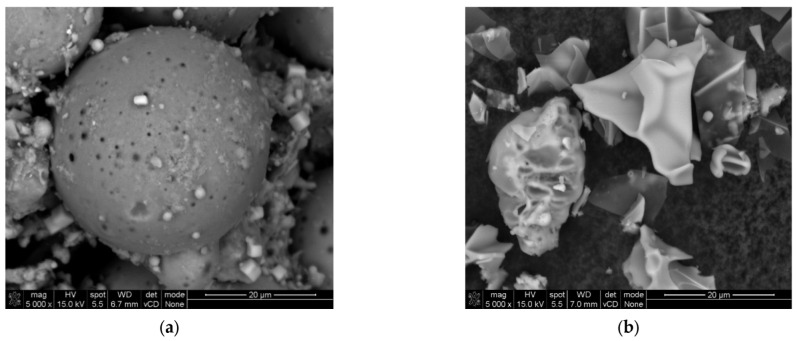
Microscopic image of (**a**) cenospheres and (**b**) perlite powder.

As part of the conducted research, six different compositions of geopolymer mortar mixes were designed, varying in their content of cenospheres (Aliorsun, Piaseczno, Poland), natural sand (EuroKrusz, Nowe Miasto, Poland), and perlite powder (JAWAR, Ciechanów, Poland). Microscopic images of cenospheres and perlite powder are shown in Figure 1. A total of 14 components were used to prepare the mixes. In one of the designed compositions which stood out from the others, methylcellulose (CFI World, Robakowo, Poland) was additionally introduced to achieve maximum compressive strength. These geopolymer mortar compositions are shown in Table 1. The precursors mix were metakaolin (Astra, Straszyn, Poland), zeolite (Astra, Straszyn, Poland), and fly ash (Siekierki CHP Plant, Warszawa, Poland). The activator mix consisted of water glass (Zakłady Chemiczne Rudniki S.A, Rudniki, Poland) and sodium hydroxide (PCC Group, Brzeg Dolny, Poland). The remaining components of the composite include redispersible powder (ELOTEX FX 2320, Celanese, Sempach Station, Switzerland) , calcium formate (Warchem, Zakręt, Poland), Chalk (Holcim, Wapienno, Poland), hydrated lime (Trzuskawica SA, Trzuskawica, Poland), tap water, and perlite sand (JAWAR, Ciechanów, Poland).

**Figure 2:**
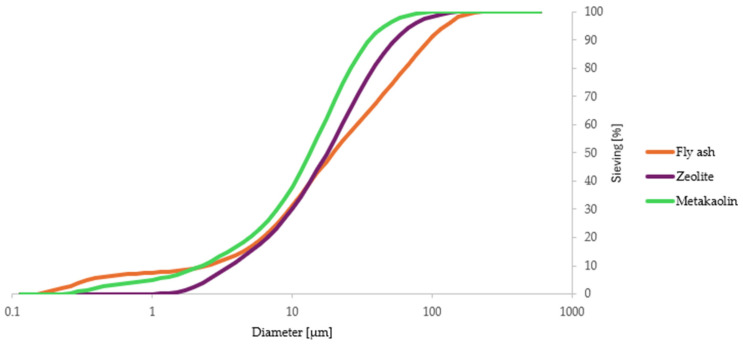
Sieve analysis results of each precursor.

**Figure 3:**
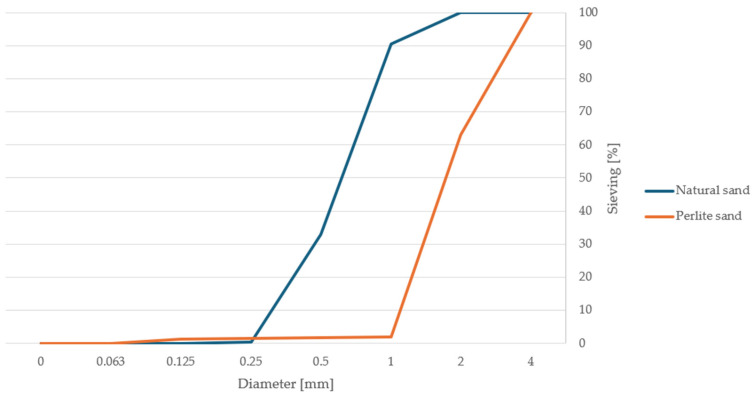
Sieve analysis results of sands.

Fly ash was analyzed using an AccuPyc helium pycnometer (Micromeritics, Norcross, GA, USA). The volume of the sample tested was 1.74 cm^3^, and the density of the sample was 2.00 g/cm^3^. The density of the metakaolin was 2.69 g/cm^3^, and that of zeolite was 2.41 g/cm^3^. Figure 2 presents the graphs resulting from the sieve analysis of each precursor. The sieve analysis was also performed for ingredients such as natural sand and perlite sand (Figure 3).

**Figure 4:**
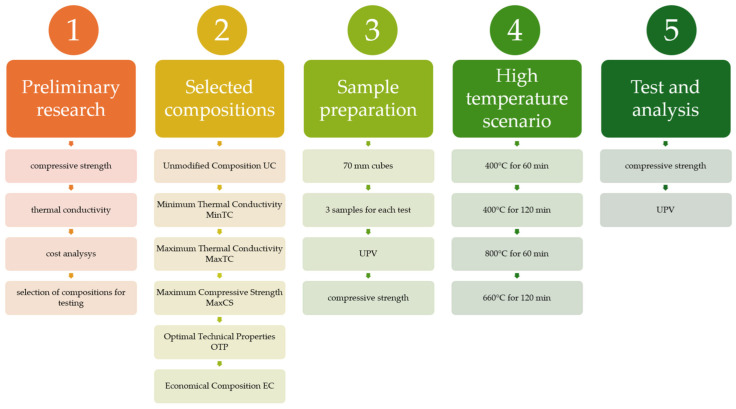
Sequence of research.

Cubic specimens with a side of 70 mm were formed for testing. The specimens were exposed to high temperatures according to different temperature rise-rate scenarios, ranging from 400 °C to 800 °C. Non-destructive UPV and destructive compressive-strength tests were performed. These tests were in accordance with standard nos. EN 12504-4 (UPV) [39] and EN 12390-3 [40] (compressive strength). The UPV is tested by Pundit device (Proceq, Schwerzenbach, Switzerland). The order in which the various tests were performed is shown in Figure 4.

**Figure 5:**
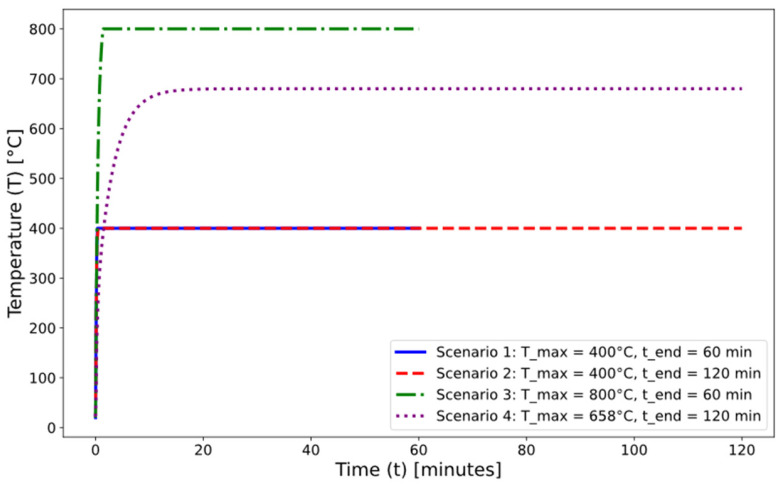
Scenarios of temperature course in the test furnace.

The design of the temperature-rise scenarios up to the planned maximum temperature was based on the cellulose fire curve (Figure 5).

**Figure 6:**
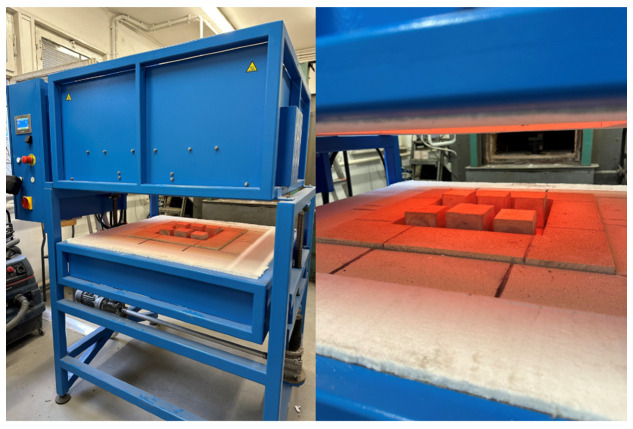
Samples in the furnace after heating.

The exposure time of the samples and the maximum temperature were varied. The first scenario involved exposing the samples to 400 °C for 60 min, the second to 400 °C for 120 min, the third to 800 °C for 60 min, and the last scenario was based on the external cladding fire curve (Figure 5). The exposure of samples to high temperatures was performed in an furnace (Figure 6).

The compositions examined in this study were chosen based on previous research, taking into account various characteristics such as thermal conductivity, compressive strength, flexural strength and density (Table 2).

The choice of individual compositions was also influenced by cost-effectiveness. Each composition is assigned a specific name and abbreviation for clarity:

- Unmodified Composition (UC): This composition serves as the control, with no modifications introduced through fine additives. It represents the baseline properties for comparison with other modified compositions.
- Minimum Thermal Conductivity (MinTC): This composition was selected for its minimal thermal conductivity coefficient (*λ*). It aims to provide the best insulation properties among the tested compositions.
- Maximum Thermal Conductivity (MaxTC): In contrast to MinTC, this composition was chosen for its Maximum Thermal Conductivity coefficient (*λ*). It helps us to understand the effect of increased thermal conductivity on the overall performance of the material.
- Maximum Compressive Strength (MaxCS): This composition was identified for its highest compressive strength (*f_c_*). It highlights the potential for enhanced load-bearing capabilities.
- Optimal Technical Properties (OTPs): This composition was selected based on its balanced technical characteristics, including thermal conductivity, compressive strength, and tensile strength. It represents the best overall technical performance.
- Economical Composition (EC): This composition takes into account not only technical performance but also the cost of materials. It represents the optimal balance between technical properties and economic efficiency.

## 3. Results

Depending on the composition of the samples, their behavior varied under Scenario 1, which involved exposure to a maximum temperature of 400 °C for 60 min. Some compositions showed only slight reductions in compressive strength, within the range of standard deviation. The composition with the highest thermal conductivity, however, experienced a 25% reduction in strength, even under this least demanding scenario. Extending the exposure time at 400 °C to 120 min did not significantly affect compressive strength, resulting in an average decrease of about 17%.

Exposure to 800 °C had a significant impact on compressive strength. The unmodified sample, which lacked additives to improve thermal insulation, did not survive the test and disintegrated. Modified samples showed compressive-strength reductions between 40% and 70%. Subjecting the samples to temperatures matching the curve for external cladding (Scenario 4) over two hours produced results comparable to the effects of exposure to 800 °C for one hour. Notably, none of the samples experienced complete destruction, and the remaining compressive strength, ranging from 2.4 to 4.1 MPa after two hours of fire exposure, would allow for the safe performance of cladding elements made from the analyzed geopolymer mortars (Table 3).

**Figure 7:**
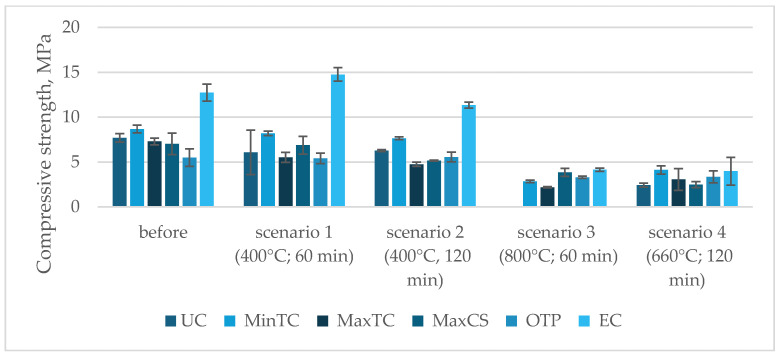
Compressive-strength test results according to scenario (MPa).

The choice of the test scenario has a significant impact on compressive strength, especially with increasing exposure temperatures. A temperature of 400 °C is a safe exposure level for the analyzed geopolymers, regardless of exposure time, as the strength reductions remained within the range of standard deviations. However, increasing the exposure temperature has a substantial effect on compressive strength, as demonstrated in Figure 7.

**Figure 8:**
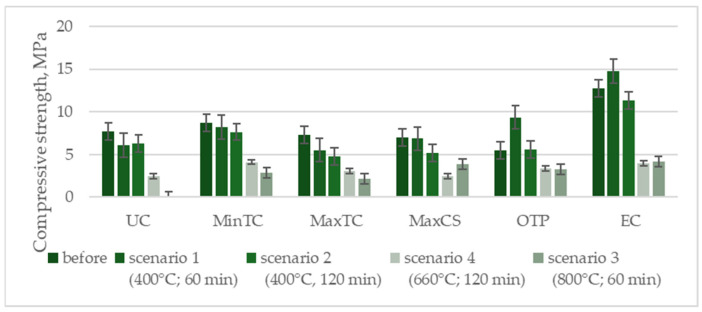
Compressive-strength test results according to composition (MPa).

Depending on their composition, the samples’ behavior varied under Scenario 1, which involved exposure to a maximum temperature of 400 °C for 60 min. Most compositions showed only slight reductions in ultrasonic pulse velocity (UPV), within the range of standard deviations. The composition with the highest thermal conductivity, however, saw a decrease of around 4%, even under this least-demanding scenario. Extending the exposure time at 400 °C to 120 min did significantly affect the UPV values, with the average reduction being from 20% to 49% (Figure 8). The graph illustrates the compressive strength (MPa) of various composite compositions before and after exposure to different temperature scenarios. The Unmodified Composition (UC) shows a significant drop in strength, especially under 800 °C (scenario 3). The Minimum Thermal Conductivity (MinTC) composition maintains moderate strength after lower temperature exposures but decreases under prolonged high temperatures. The Maximum Thermal Conductivity (MaxTC) composition follows a similar pattern, with notable reductions after exposure. The Maximum Compressive Strength (MaxCS) composition, which starts with the highest strength, retains a relatively good performance across all scenarios. The Optimal Technical Property (OTP) composition, balanced for both thermal and mechanical properties, suffers considerable strength loss, particularly at higher temperatures. The Economical Composition (EC) begins with the highest initial strength and retains a substantial portion of it, even after exposure to elevated temperatures, though it also shows a drop at 800 °C. Overall, all compositions experience strength reduction with increasing temperatures, with MaxCS and EC performing better under extreme conditions.

It is important to note that the reduction in ultrasonic pulse velocity (UPV) indicates changes in the internal structure of the composite before it significantly affects compressive strength. This suggests that UPV measurements can serve as an early indicator of structural degradation, providing insights into material weakening before substantial losses in compressive strength are observed. Exposure to 800 °C had a significant impact on UPV. The unmodified sample, which lacked thermal insulation additives, experienced a drastic reduction, with its UPV dropping by nearly 70%. Modified samples showed decreases in UPV ranging from 40% to 60%. This is analogous to changes in compressive strength. When the samples were subjected to temperatures following the curve for external cladding (Scenario 4) for two hours, the results were comparable to those seen after exposure to 800 °C for one hour. None of the samples experienced total structural failure, and the remaining UPV values, ranging from 747 to 1149 m/s after two hours of fire exposure (Table 4).

**Figure 9:**
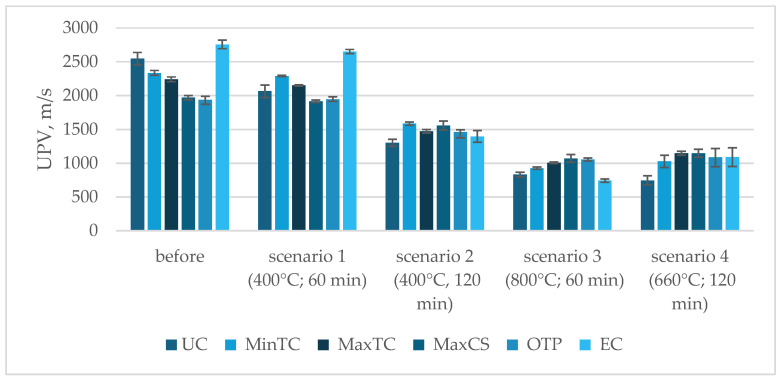
UPV results in dependance of scenario (m/s).

The nature of changes in ultrasonic pulse velocity (UPV) is analogous to the changes in compressive strength depending on the test scenario. However, it is important to note that non-destructive testing may reveal structural changes that do not necessarily impact compressive strength. While UPV provides valuable insights into structural integrity, these changes might not always correlate directly with measurable reductions in compressive strength (Figure 9).

**Figure 10:**
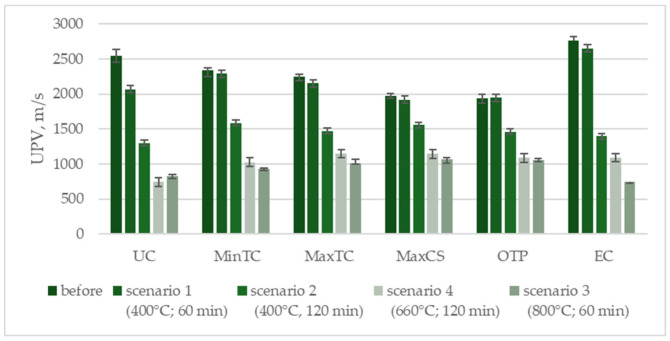
UPV results in dependance of composition (m/s).

The UPV analyses closely align with the compressive-strength results. Compositions modified with perlite powder and cenospheres demonstrated greater resistance to high temperatures. Even a slight modification of the mix with these additives prevented any reduction in wave velocity after 60 min at 400 °C. Notably, the thermal conductivity of the composites (MinTC and MaxTC) did not significantly impact wave velocity. Compositions with higher overall dosages of the additives, such as MinTC, MaxTC, MaxCS, and OPT, experienced smaller reductions in wave velocity. In contrast, the composition with a lower dosage of these additives (EC), despite having the highest initial wave velocity, showed the largest percentage decreases—up to 70% (Figure 10).

There is a general trend showing a positive correlation between wave velocity (V, m/s) and compressive strength (fc, MPa). Higher wave velocities tend to correspond with higher compressive strengths. For instance, samples with higher velocities, like 2547 m/s and 2757 m/s, exhibit compressive strengths of 7.7 MPa and 12.7 MPa, respectively. Conversely, lower wave velocities, such as 831 m/s and 927 m/s, are associated with significantly lower compressive strengths, with values as low as 0 MPa and 2.8 MPa, respectively.

**Figure 11:**
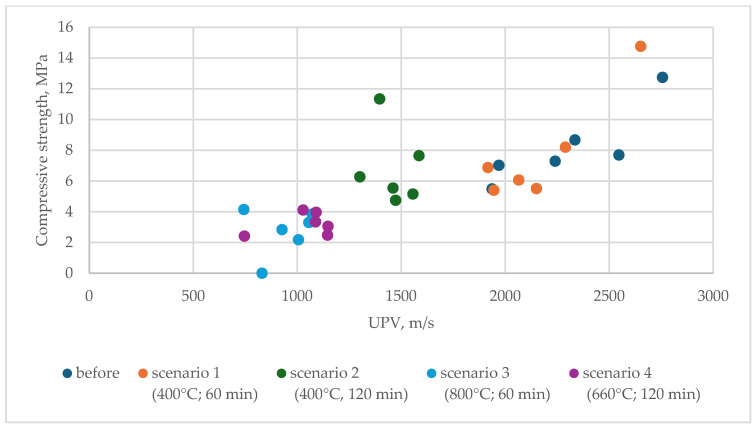
Relationship between ultrasonic pulse velocity and compressive strength.

However, there are exceptions, such as wave velocities around 1461–1473 m/s, where compressive strengths vary more widely (from 4.7 MPa to 11.4 MPa), suggesting that other factors might influence the strength at certain points. In general, though, the data support the idea that a higher wave velocity generally indicates a higher compressive strength, reinforcing the use of non-destructive testing like UPV (ultrasonic pulse velocity) for estimating compressive strength. Additionally, with an estimated accuracy of approximately ±1 MPa, compressive strength can be reasonably estimated based on non-destructive testing (Figure 11). This finding has practical implications, as such a correlation can be useful for assessing the level of damage to geopolymer elements after a fire without the need for core sampling and further damage to the structure.

## 4. Discussion

The findings from our study on geopolymer samples with various modifiers show both similarities and differences compared to the publications related to eco-concrete’s high-temperature resistance.

Our results align with the observation that eco-concrete generally maintains compressive strength up to 600–700 °C due to matrix densification [34], with significant reductions occurring above 700 °C due to thermal decomposition. Similarly, tested geopolymer samples experienced substantial reductions in compressive strength and ultrasonic pulse velocity (UPV) under high temperatures, with unmodified samples disintegrating at 800 °C and modified samples showing strength reductions of 40% to 70%.

**Figure 12:**
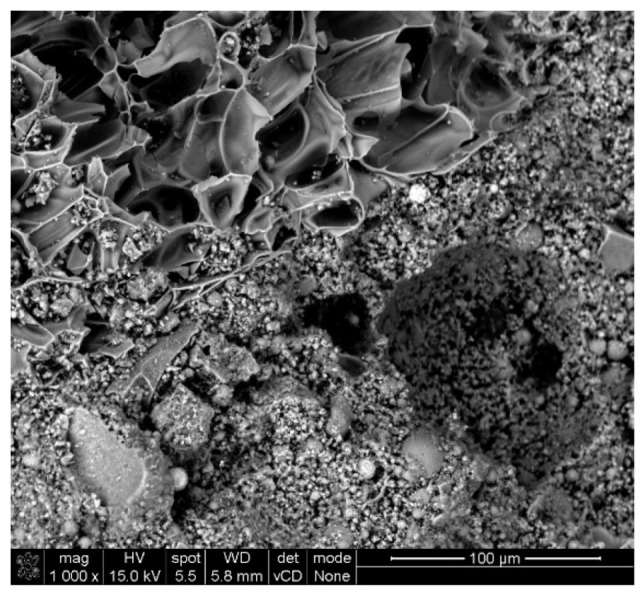
Microscopic image of a geopolymer sample with analyzed modifiers.

The eco-concretes improve matrix adhesion and mechanical properties, leading to better performance compared to ordinary Portland cement (OPC) concrete [34]. In the case of the analyzed geopolymers, an amorphous and dense matrix structure was observed, despite the presence of porous particles of perlite sand, perlite powder, and cenospheres within it (Figure 12).

This confirms the impact of the matrix structure on high-temperature resistance [30,31]. Although our study did not directly address sintering, the observed changes in UPV and compressive strength in geopolymer samples suggest that high thermal conductivity and exposure conditions affect structural integrity.

Regarding spalling and cracking, the summary indicates that eco-concrete generally performs better than OPC concrete under high temperatures, with color changes after fire exposure not necessarily indicating reduced structural integrity. Our study also showed significant reductions in UPV and compressive strength in geopolymer samples, though spalling was not explicitly reported.

Finally, while it is noted that rapid cooling can cause thermal stress and cracking in eco-concrete [34], our study did not specifically address the cooling effects. However, the overall impact of high temperatures on geopolymer samples underscores the importance of material composition and thermal exposure in determining performance.

Future research should also focus on investigating the long-term resistance of the modified composites containing highly porous additives. However, this is unlikely to have a significant impact, as the matrix plays a key role in determining the material’s performance under thermal cycling. As demonstrated in the literature study [18,19,20], the matrix has shown strong resistance to repeated thermal exposure, suggesting that, even with the incorporation of porous additives, the overall structural integrity of the composites would remain stable. Nonetheless, further tests are necessary to confirm this hypothesis and to assess the durability under extended high-temperature conditions.

## 5. Conclusions

The investigation into the performance of geopolymer samples with various modifiers under different temperature scenarios has provided significant insights into their structural integrity and thermal resistance. Our findings indicate the following:

- Compressive strength and ultrasonic pulse velocity (UPV): The compressive strength and UPV of geopolymer samples demonstrated a clear dependence on the exposure temperature and duration. At a maximum temperature of 400 °C for 60 min, most compositions showed only minor reductions in compressive strength and UPV, consistent with the range of standard deviations. However, increasing the temperature to 800 °C caused substantial decreases in both metrics, with unmodified samples failing completely. Modified samples exhibited strength reductions between 40% and 70%, highlighting the impact of temperature on material performance.
- Analogies to eco-concrete: The results are analogous to those observed in eco-concrete studies, which also show that compressive strength and structural integrity are maintained up to certain temperatures due to matrix densification. However, beyond these temperatures, significant degradation occurs. Similar to eco-concrete, geopolymer samples exhibited a reduced performance at high temperatures, with changes in UPV indicating structural degradation before significant losses in compressive strength.
- Practical implications: The correlation between UPV and compressive strength suggests that non-destructive testing methods can effectively estimate compressive strength based on structural changes. This can be particularly useful for assessing damage levels in geopolymer components post-fire, without the need for invasive sampling.

Overall, these findings emphasize the importance of material composition and structural integrity in determining the high-temperature performance of geopolymers. The study provides insights into how different modifiers and exposure conditions impact the thermal resistance of geopolymer materials, with practical implications for their use in high-temperature applications.

The presented study highlights the potential application of the composite as a material for cladding in sustainable buildings. The composite exhibits significant thermal insulation properties, ensures safety under fire conditions by retaining a large portion of its strength, and does not show splitting effects. In addition to being largely composed of waste materials, it can contribute to reducing the carbon footprint both during construction and operation by limiting heat transfer between building partitions. Another ecological advantage is the material’s ability to retain its properties after exposure to temperatures up to 400 °C. This means that it would not need to be replaced after such exposure, thus minimizing waste generation.

## Figures and Tables

**Table 1:** Composition of composites per cubic meter (kg).

Composition	Metakaolin	Zeolite	Fly Ash	Redispersible Powder	Methylcellulose	Calcium Formate	Natural Sand	Chalk	Hydrated Lime	Sodium Hydroxide (NaOH)	Water Glass (Sodium Silicate)	Water (H_2_O)	Perlite Sand	Perlite Powder	Cenospheres
UC *	50	50	400	10	0	5	880	60	50	397	80	84	30	0	0
MinTC *	50	50	400	10	0	5	705	60	50	397	80	84	30	40	40
MaxTC *	50	50	400	10	0	5	636	60	50	397	80	84	30	5	75
MaxCS *	50	50	400	10	4	5	663	60	50	397	80	84	30	75	40
OTPs *	50	50	400	10	0	5	597	60	50	397	80	84	30	65	65
EC *	50	50	400	10	0	5	816	60	50	397	80	84	30	40	5

* Unmodified Composition (UC); Minimum Thermal Conductivity (MinTC); Maximum Thermal Conductivity (MaxTC); Maximum Compressive Strength (MaxCS); Optimal Technical Properties (OTPs); Economical Composition (EC).

**Table 2:** Compressive strength and flexural strength after 14 days, thermal conductivity coefficient and density test results.

Composition	Flexural Strength *f_t_*, MPa	Compressive Strength *f_c_*, MPa	Thermal Conductivity *λ*, W/(m·K)	Density kg/m^3^
UC *	1.8	4.6	0.2410	1540
MinTC *	1.6	3.5	0.1836	1510
MaxTC *	1.9	5.3	0.3127	1450
MaxCS *	1.9	6.7	0.2622	1540
OTPs *	1.6	5.6	0.2374	1480
EC *	1.6	5.0	0.2557	1580

* Unmodified Composition (UC); Minimum Thermal Conductivity (MinTC); Maximum Thermal Conductivity (MaxTC); Maximum Compressive Strength (MaxCS); Optimal Technical Properties (OTPs); Economical Composition (EC).

**Table 3:** Compressive-strength test results (MPa).

Composition	Before	After 1st Scenario	After 2nd Scenario	After 3th Scenario	After 4th Scenario
UC *	7.7 ± 0.5	6.1 ± 2.5	6.3 ± 0.1	0.0 ± 0.0	2.4 ± 0.2
MinTC *	8.7 ± 0.4	8.2 ± 0.2	7.6 ± 0.1	2.8 ± 0.1	4.1 ± 0.5
MaxTC *	7.3 ± 0.4	5.5 ± 0.6	4.7 ± 0.2	2.2 ± 0.1	3.1 ± 1.2
MaxCS *	7.0 ± 1.2	6.9 ± 1.0	5.2 ± 0.1	3.9 ± 0.4	2.5 ± 0.3
OTPs *	5.5 ± 1.0	5.4 ± 0.6	5.5 ± 0.5	3.3 ± 0.1	3.3 ± 0.7
EC *	12.7 ± 0.9	14.8 ± 0.8	11.3 ± 0.3	4.1 ± 0.2	4.0 ± 1.5

* Unmodified Composition (UC); Minimum Thermal Conductivity (MinTC); Maximum Thermal Conductivity (MaxTC); Maximum Compressive Strength (MaxCS); Optimal Technical Properties (OTPs); Economical Composition (EC).

**Table 4:** UPV results (m/s).

Composition	Before	After 1st Scenario	After 2nd Scenario	After 3th Scenario	After 4th Scenario
UC *	2547 ± 91	2065 ± 93	1302 ± 52	831 ± 35	747 ± 67
MinTC *	2336 ± 36	2290 ± 10	1586 ± 24	927 ± 15	1028 ± 90
MaxTC *	2241 ± 35	2151 ± 11	1474 ± 27	1007 ± 10	1149 ± 30
MaxCS *	1970 ± 32	1917 ± 18	1557 ± 67	1072 ± 58	1146 ± 59
OTPs *	1937 ± 53	1946 ± 38	1461 ± 33	1056 ± 22	1088 ± 131
EC *	2727 ± 64	2652 ± 31	1397 ± 86	743 ± 23	1091 ± 139

* Unmodified Composition (UC); Minimum Thermal Conductivity (MinTC); Maximum Thermal Conductivity (MaxTC); Maximum Compressive Strength (MaxCS); Optimal Technical Properties (OTPs); Economical Composition (EC).

## Data Availability

The original contributions presented in the study are included in the article, further inquiries can be directed to the author.
